# Calcineurin Inhibitor CN585 Exhibits Off-Target Effects in the Human Fungal Pathogen *Aspergillus fumigatus*

**DOI:** 10.3390/jof8121281

**Published:** 2022-12-07

**Authors:** Praveen R. Juvvadi, Benjamin G. Bobay, D. Christopher Cole, Monaf Awwa, William J. Steinbach

**Affiliations:** 1 Department of Pediatrics, Arkansas Children’s Research Institute, University of Arkansas for Medical Sciences, Little Rock, AR 72202, USA; 2 Duke University NMR Center, Duke University Medical Center, Durham, NC 27710, USA; 3 Department of Biochemistry, Duke University Medical Center, Durham, NC 27710, USA; 4 Department of Radiology, Duke University Medical Center, Durham, NC 27710, USA; 5 Department of Pediatrics, Duke University Medical Center, Durham, NC 27710, USA; 6 Department of Chemistry, Stony Brook University, Stony Brook, NY 11794, USA; 7 Department of Pediatrics, Arkansas Children’s Hospital, University of Arkansas for Medical Sciences, Little Rock, AR 72202, USA

**Keywords:** *Aspergillus*, calcineurin, CN585, cyclosporin A, cyclophilin, FK506, FKBP12, molecular dynamics, molecular docking

## Abstract

Calcineurin (CN) is an attractive antifungal target as it is critical for growth, stress response, drug resistance, and virulence in fungal pathogens. The immunosuppressive drugs, tacrolimus (FK506) and cyclosporin A (CsA), are fungistatic and specifically inhibit CN through binding to their respective immunophilins, FK506-binding protein (FKBP12), and cyclophilin (CypA). We are focused on CN structure-based approaches for the development of non-immunosuppressive FK506 analogs as antifungal therapeutics. Here, we examined the effect of the novel CN inhibitor, CN585, on the growth of the human pathogen *Aspergillus fumigatus*, the most common cause of invasive aspergillosis. Unexpectedly, in contrast to FK506, CN585 exhibited off-target effect on *A. fumigatus* wild-type and the azole- and echinocandin-resistant strains. Unlike with FK506 and CsA, the *A. fumigatus* CN, FKBP12, CypA mutants (Δ*cnaA*, Δ*fkbp12*, Δ*cypA*) and various FK506-resistant mutants were all sensitive to CN585. Furthermore, in contrast to FK506 the cytosolic to nuclear translocation of the CN-dependent transcription factor (CrzA-GFP) was not inhibited by CN585. Molecular docking of CN585 onto human and *A. fumigatus* CN complexes revealed differential potential binding sites between human CN versus *A. fumigatus* CN. Our results indicate CN585 may be a non-specific inhibitor of CN with a yet undefined antifungal mechanism of activity.

## 1. Introduction

Calcineurin (CN) is a highly conserved Ca^2+^-calmodulin-dependent serine/threonine phosphatase in mammalian and fungal systems [1,2]. Apart from its multi-functional cellular roles in mammalian systems [3,4,5], its importance for regulating growth, stress responses, and virulence in different fungal pathogens is well established in species of *Aspergillus*, *Candida*, *Cryptococcus*, and *Mucor* [1,6].

CN functions as a complex of the catalytic subunit (CnA) and regulatory subunit (CnB) and is activated by increased intracellular Ca^2+^ concentration and the binding of the Ca^2+^-binding protein calmodulin [7]. Members of the NFAT transcription factor family in their hyperphosphorylated form are the major substrates dephosphorylated by CN in immune cells [8,9]. Following dephosphorylation, nuclear translocation of NFAT and its binding onto regulatory promoter elements of genes stimulates the expression of cytokines and chemokines, which play vital role in the initiation of immune responses [10]. The well-established mode of CN-complex inhibition is through the immunosuppressive drugs, tacrolimus (FK506), and cyclosporin A (CsA), that are widely used in transplantation to prevent graft rejection [9,11,12,13]. FK506 and CsA bind to their respective immunophilins, FKBP12 (12-kDa FK506 binding protein), and CypA (cyclophilin A). This immunophilin-immunosuppressant complex then binds CN complex to block substrate binding and inhibit CN function [14,15].

Although FK506 and CsA exhibit potent antifungal activity in vitro, their host immunosuppressive effects make it difficult to exploit them as antifungal therapeutics. In an effort to develop CN inhibitors that could be used as antifungals, FK506 analogs that permeated mammalian cells but not fungal cells were synthesized and dosed along with FK506 to antagonize the immunosuppressive effect of FK506 without lowering its antifungal activity [16]. Non-immunosuppressive FK506 analogs with antifungal activity have also been generated through combinatorial biosynthetic approaches with structural modifications at CN-binding and FKBP-binding regions [17,18]. Our recent studies have focused on structural analysis of fungal CN-inhibitor complexes to identify differences between mammalian and fungal CN ternary complexes to enable the rational design of fungal-specific non-immunosuppressive FK506 analogs [19,20,21]. Here, we tested the inhibitory activity of one novel mammalian CN inhibitor, CN585 [22], on the fungal pathogen *A. fumigatus*. CN585 is a cell permeable 2,6-diaryl-substituted pyrimidine derivative belonging to a new class of CN inhibitors and characterized as an immunosuppressive and non-competitive reversible inhibitor of CN in mammalian cells. It has been shown to specifically inhibit mammalian CN activity without affecting other serine/threonine phosphatases [22].

## 2. Materials and Methods

### 2.1. Strains, Culture Conditions and In Vitro Drug Susceptibility Assays

The *A. fumigatus* wild-type strain (*akuB*^KU80^), azole-resistant clinical isolates (F14946 and F16216), echinocandin-resistant strain (EMFR-S678P), CN pathway deletion strains including CN catalytic subunit deletion (Δ*cnaA*), immunophilin FKBP12 and cyclophilin A deletion strains (Δ*fkbp12*; Δ*cypA*) and various *cnaA* mutated strains were utilized for antifungal susceptibility assays. Strains were cultured on GMM (glucose minimal medium) agar for 5 days at 37 °C for collection of conidia for the assays. Broth dilution antifungal susceptibility testing was performed according to the Clinical Laboratory Standards Institute (CLSI) guidelines in RPMI-1640 medium with slight modifications. Conidia were harvested in 0.05% Tween 80 and counted to obtain a spore concentration of 10^4^/mL and subsequently diluted to obtain 5 × 10^2^/mL in each well (200 µL) of a 96-well plate. Effect of CN585 (Sigma-Aldrich, St. Louis, MO, USA) or FK506 (Astellas Pharma, Tokyo, Japan) on growth was tested at concentrations ranging from 0 to 10 µg/mL. Interpretation of results on growth inhibition was performed after 48 h of growth at 37 °C by microscopic observation. Bright-field photomicrographs were acquired on a Nikon Diaphot phase contrast inverted microscope equipped with a Canon T5i digital camera.

### 2.2. Microscopy

To assess the effect of CN585 or FK506 on CrzA localization, conidia from the *A. fumigatus akuB*^KU80^ strain expressing CrzA-GFP fusion protein [23] were cultured for 18 h at 37 °C in 35 mm cover glass bottom Petri dish (Matek) in a total volume of 1000 µL GMM broth. After 18 h of growth, cultures were treated with FK506 (0.5 µg/mL) or CN585 (6 µg/mL) for 30 min and 120 min. Following CN inhibitor treatments, the respective cultures were exposed to CaCl_2_ (50 mM) for time points ranging between 0–60 min and visualized by fluorescence microscopy using an Axio Observer 3 scope. Differential interference contrast (DIC) and GFP-fluorescent images were captured at 10 min intervals over a period of 1 h using the 100x/1.4 oil Plan Apochromat DIC objective and Chroma GFP filter with Zeiss Axiocam 506 mono high resolution digital camera.

### 2.3. Molecular Modeling of Aspergillus fumigatus eIF4E

*A. fumigatus* eIF4E has not been structurally characterized, therefore, the protein sequence of *A. fumigatus* eIF4E (XP_748025.1) was submitted to the SWISS-MODEL web server to develop a structural model [24]. The sequence aligned well with that of the “Complex crystal structure of *Ascaris suum* eIF4E-3 with m^7^G cap” (PDB code: 3M93) and this was used as a template for the construction of the *A. fumigatus* eIF4E model [25].

### 2.4. Molecular Dynamic Simulations

All molecular dynamic (MD) simulations were performed with the GROMACS 2020 software package utilizing six CPU cores and one NVIDIA Tesla K80 GPU to provide a solvated representation (Abraham MJ, van der Spoel D, Lindahl E, Hess B. Gromacs Development Team. *GROMACS User Manual*, ver. 5.0, 2019). The single starting conformations used for all MD simulations were the X-ray crystal structures 6TZ7 (crystal structure of *A. fumigatus* CnA, CnB, FKBP12 and FK506 with FKBP12 and FK506 removed), 1MF8 (crystal structure of human CN complexed with CsA and human cyclophilin) with CsA and cyclophilin removed, and a structural model of *A. fumigatus* eIF4E (see Section 2.3) [26,27]. MD simulations were performed with the AMBER 99sb-ildn force field using the flexible simple point charge water model. The initial structures were immersed in a periodic water box with a dodecahedral shape that extended 1 nm beyond the protein in any dimension and neutralized with counter ions. Energy minimization was accomplished using the steepest descent algorithm with a final maximum force of <100 kJ mol^−1^ min^−1^ (0.01 nm step size, cutoff of 1.0 nm for the neighbor list, Coulomb interactions, and van der Waals interactions). After energy minimization, the system was subjected to equilibration at 300 K and normal pressure for 1 ns. All bonds were constrained with the LINCS algorithm, and virtual sites were used to allow a 4 fs time step (cutoff of 1.0 nm for the neighbor list, Coulomb interactions, and van der Waals interactions). After temperature stabilization, pressure stabilization was achieved by utilizing the v-rescale thermostat to hold the temperature at 300 K and the Berendsen barostat was used to bring the system to 1 bar pressure. Production MD calculations (100 ns) were performed under the same conditions, except that the positional restraints were removed (cutoff of 1.0 nm for the neighbor list, Coulomb interactions, and van der Waals interactions). GROMACS built-in and homemade scripts were used to analyze the MD simulation results. The α-carbon root-mean-square deviation (Cα-RMSD), radius of gyration (R_g_), and center of mass (COM) were analyzed to confirm the stability and accuracy of the MD simulations. All images were produced in part through PyMOL (*The PyMOL Molecular Graphics System*, ver. 2.0; Schrödinger, LLC., New York, NY, USA).

### 2.5. Molecular Docking

Default High Ambiguity Drive Docking (HADDOCK) parameters were used throughout the docking procedure on the webserver with the following exceptions: auto_passive_radius = 6.5, rotate180_0 = False, crossdock = True, and calcdesolv = True [28,29]. Every 9 ns frame after equilibration (10 ns) from the MD simulation of the protein was extracted to form an ensemble of structures prior to docking over the last 90 ns. This helps to ensure the docking procedure examines multiple conformational states within the potential binding site prior to each docking event and the docked structures, providing a more accurate representation of the docked structures and their conformational flexibility in solution. Active residues for the proteins were defined as residues with a ≥60% solvent exposure for the protein and 100% of the CN585 small molecule was set to as actively involved in the interaction. The HADDOCK webserver was used for docking, therefore, the CN585 small molecule topology and parameter file was automatically generated through PRODRG [30]. Through the HADDOCK webserver, one thousand structures were generated for the first iteration (rigid docking), and 400 were generated for each subsequent iteration (semiflexible docking and water refinement). The Cα-RMSD values of the complexes were calculated using ProFit version 3.1 (SciTech Software, Chico, CA, USA). The structures were clustered based on Cα-RMSD and scored using HADDOCK’s internal scoring mechanism. The resulting Z-score analysis provided by HADDOCK indicates how many standard deviations from the average this cluster is located in terms of score (the more negative the better). The top cluster is the most reliable according to HADDOCK. The structures that represented the median score for the most reliable cluster were then visualized with PyMOL to ensure the integrity of the complex formed (The PyMOL Molecular Graphics System, Version 2.0 Schrödinger, LLC.).

## 3. Results

### 3.1. CN585 Significantly Inhibits Growth of A. fumigatus Wild-Type and the Azole- and Echinocandin-Resistant Strains

**Figure 1:**
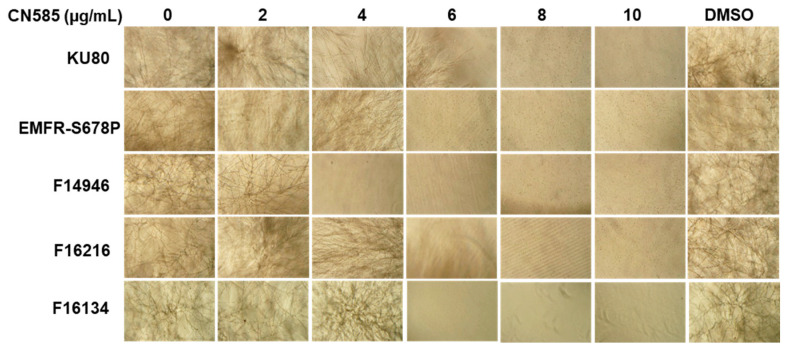
Growth of the wild-type *A. fumigatus* (KU80) and the echinocandin-resistant (EMFR-S678P) and the azole-resistant (F14946, F16216 and F16134) strains in the presence of increasing concentrations of CN585. DMSO control growth is shown. Growth was monitored for 2 days in RPMI at 37 °C. Light microscopy photographs (×10 magnification) after 2 days of growth are shown. Note the complete inhibition in growth at >8 µg/mL CN585. Growth susceptibility assays were performed three times in triplicate.

Both FK506 and CsA inhibit *A. fumigatus* growth by inducing the formation of hyperbranched and stunted hyphae with aberrant septation mimicking a CN deletion phenotype [31,32]. As a first step to analyze the effect of CN585 on *A. fumigatus* growth, the wild-type (*akuB*^KU80^) strain was cultured in RPMI liquid medium at increasing concentrations of CN585 ranging from 0–10 µg/mL based on the reported IC_50_ value for mammalian CN inhibition [22]. In contrast to our previously observed growth phenotypes following FK506/CsA treatment, CN585 treatment did not result in any hyperbranching, hyperseptation, or a stunted growth phenotype (Figure 1). CN585 caused a concentration-dependent growth inhibition of hyphal growth and, unlike FK506 or CsA, resulted in complete growth inhibition between 6–8 µg/mL. Azoles and echinocandins are currently used drugs for the treatment of invasive aspergillosis and target the fungal cell membrane (ergosterol) and cell wall (β-glucan and chitin), respectively. Strains with mutations in the *cyp51A* gene (F14946 and F16216) and *fksA* gene (EMFR-S678P) exhibit resistance to azoles and echinocandins, respectively. To further identify any strain-dependent variations in susceptibility to CN585 we next tested the *A. fumigatus* azole- (F14946 and F16216) [33] and echinocandin-resistant (EMFR-S678P) [34] strains which also showed a similar trend in susceptibility.

### 3.2. CN585-Mediated Growth Inhibition Is Independent of Key Residues in the CN-Effector Binding Regions

**Figure 2:**
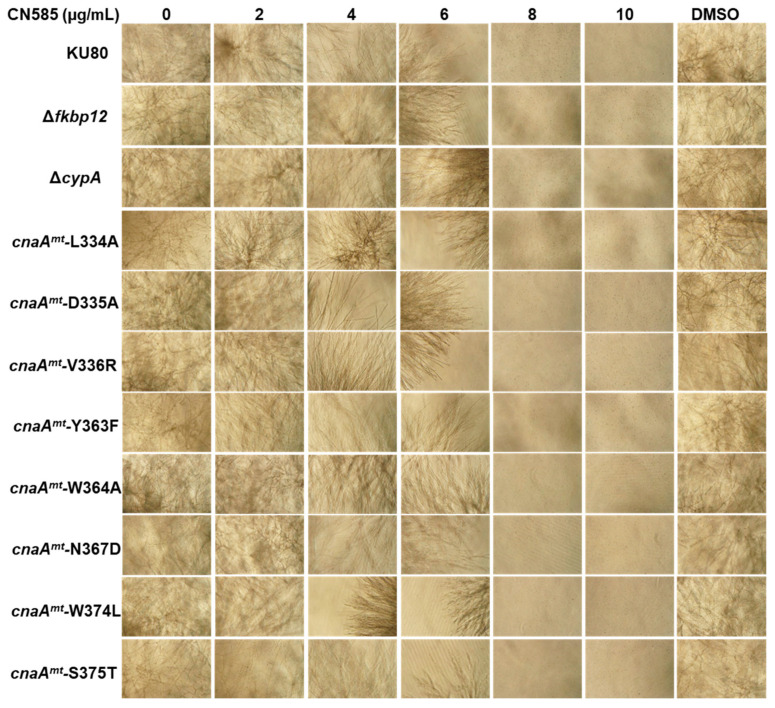
Growth of the wild-type *A. fumigatus* (KU80), FK506-binding protein deletion (Δ*fkbp12*), cyclophilin A deletion (*ΔcypA*) and the various *cnaA* mutant strains in the presence of increasing concentrations of CN585. DMSO control growth is shown. Growth was monitored for 2 days in RPMI at 37 °C. Light microscopy photographs (×10 magnification) after 2 days of growth are shown. Note the complete inhibition in growth for all the strains at >8 µg/mL CN585. Growth susceptibility assays were performed twice in triplicate.

Previous analysis of human CN inhibition by CN585 indicated that CsA-Cyp18 (cyclosporin A-cyclophilin) complex and CN585 may share similar binding site on the CN catalytic subunit (CnA) [22]. Earlier studies have also shown that the loop 7 region in CnA that is in close proximity to the CsA-CypA and FKBP12-FK506 complex binding region influences CN activity [35]. Based on our *A. fumigatus* CN ternary complex structure, the CnA loop 7 region (residues 328–339) is proximal to the FK506-FKBP12 complex binding region. In order to verify if the mode of CN inhibition by CN585 is mediated through interaction with any of these key residues near the catalytic domain including the loop 7 region or in the CnB-binding helix, which includes the CsA-CypA binding and the FK506-FKBP12 binding domains, we next tested the susceptibility of various CnA mutant strains to CN585. However, none of the loop 7 residue (Leu334; Asp335; Val336) mutants (*cnaA^mt^-*L334A; *cnaA^mt^-*D335A; *cnaA^mt^-*V336R) induced any resistance to CN585 (Figure 2) but exhibited variable resistance to CsA at 10 µg/mL (Figure S1). Mutations in residues Tyr363 (*cnaA^mt^-*Y363F) and Trp364 (*cnaA^mt^-*W364A) which are present in the CsA-CypA binding region also did not have any impact on CN585 sensitivity but exhibited resistance to CsA at 10 µg/mL (Figure S1). Mutations in the critical residues Asn367, Trp374 and Ser375 (*cnaA^mt^-*N367D; *cnaA^mt^-*W374L; *cnaA^mt^-*S375T), known to influence FK506 sensitivity [36], also did not reveal any altered sensitivity to CN585.

To further investigate the direct role of immunophilins in binding to CN585 and inhibiting CN, we next generated a cyclophilin A deletion strain (Δ*cypA*) (Figure S2) and also utilized the FKBP12 (Δ*fkbp12*) deletion strain [37] for susceptibility testing. While the Δ*cypA* and the Δ*fkbp12* strains were resistant to CsA and FK506, respectively, no induction in resistance to CN585 was noted in the Δ*cypA* or the Δ*fkbp12* strains.

### 3.3. Hypersensitivity of the A. fumigatus CN Deletion Mutant to CN585 Indicates Off-Target Effects of CN585

**Figure 3:**
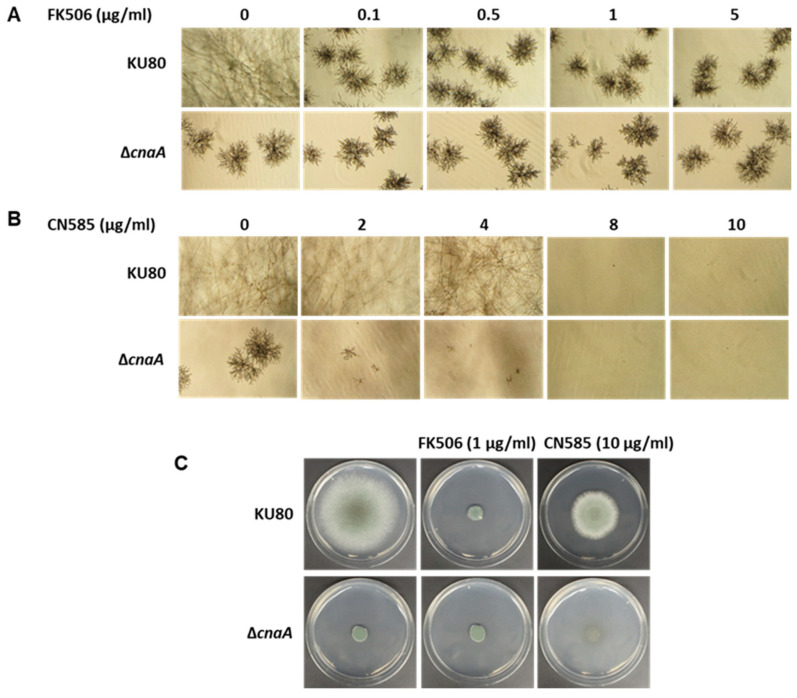
(**A**) Growth of the wild-type *A. fumigatus* (KU80) and the CnA deletion (Δ*cnaA*) strains in the presence of increasing concentrations of FK506. Note that the Δ*cnaA* strain is not susceptible to growth inhibition by FK506. (**B**) Growth of the wild-type *A. fumigatus* (KU80) and the CnA deletion (Δ*cnaA*) strains in the presence of increasing concentrations of CN585. Note that the Δ*cnaA* strain is sensitive to CN585 with complete inhibition in growth at >4 µg/mL CN585. Growth was monitored for 2 days in RPMI at 37 °C. Light microscopy photographs (×10 magnification) after 2 days of growth are shown. (**C**) Comparative growth of the wild-type *A. fumigatus* (KU80) and the CnA deletion (Δ*cnaA*) strains in the presence of FK506 and CN585 on GMM agar for 5 days at 37 °C. Note that the Δ*cnaA* strain is sensitive to CN585 with complete inhibition in growth at 10 µg/mL CN585 in contrast to FK506. Growth susceptibility assays were performed in triplicate.

In order to test if CN585 specifically inhibited CN function in *A. fumigatus*, we next compared the susceptibility of the *A. fumigatus* CN deletion strain (Δ*cnaA*) [38] to both FK506 and CN585 in RPMI liquid medium for 48 h. While FK506 treatment of the wild-type strain mimicked the CN deletion phenotype, the CN deletion strain was not susceptible to inhibition by FK506 treatment in a range of concentration between 0.1–5 µg/mL (Figure 3A) indicating the absence of target for FK506. Treatment with >5 µg/mL of FK506 also yielded a similar pattern, further confirming the specific action of FK506 on CN. However, the CN deletion strain exhibited a concentration-dependent growth inhibition with CN585 (Figure 3B). The CN deletion strain exhibited drastic reduction in growth at 2 µg/mL of CN585 with almost complete growth inhibition at ≥4 µg/mL CN585. To further examine if CN585 is causing a fungicidal effect, we cultured the wild-type and CN deletion strains on GMM agar medium for 5 days. While CN585 at 10 µg/mL could not mimic the CN inhibition phenotype observed with FK506 at 1 µg/mL in the wild-type strain, there was a drastic growth inhibition of the CN deletion strain in the presence of CN585 (10 µg/mL) as opposed to no inhibition in growth in the presence of FK506 (1 µg/mL) (Figure 3C). These results indicated the possibility of CN585 having off-target antifungal effects in *A. fumigatus*.

### 3.4. CN585-Mediated Growth Inhibition Is Independent of the CN-Dependent Transcription Factor CrzA

**Figure 4:**
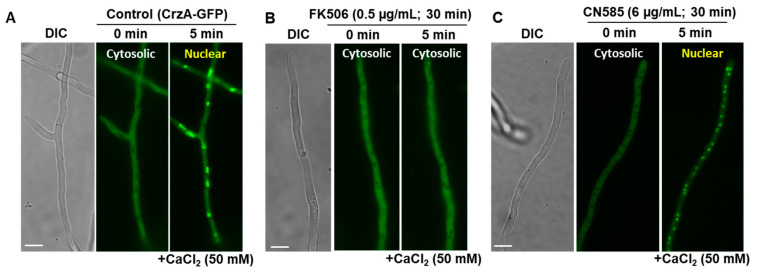
Microscopic localization CrzA-GFP in *A. fumigatus* in the absence or presence of FK506 and CN585. (**A**) Nuclear localization of CrzA-GFP under control conditions. (**B**) Inhibition of nuclear localization of CrzA by FK506. (**C**) Nuclear localization of CrzA-GFP under CN585 treated conditions. DIC-Differential Interference Contrast; Scale bar, 10 µm.

CN585 was shown to inhibit nuclear translocation of GFP-NFAT in HeLa cells [22]. As Crz1 is a well-known NFAT ortholog in fungi [39,40], we utilized an *A. fumigatus* strain expressing CrzA-GFP fusion protein to verify the effect of CN585 on CrzA’s localization in vivo. Cultures grown for 20 h under control conditions were incubated with 6 µg/mL CN585 for various time periods (10, 30 and 120 min, respectively) and stimulated by the addition of 50 mM CaCl_2_ for 5–60 min. Simultaneously, FK506 treatment (100–500 ng/mL) was also performed as a comparison. While the addition of CaCl_2_ under control conditions resulted in nuclear translocation of CrzA-GFP within 5 min (Figure 4A), the addition of FK506 blocked CrzA’s nuclear translocation indicating the inhibition of CN signaling by FK506 (Figure 4B). However, CN585 did not have any impact on the nuclear localization of CrzA (Figure 4C), further indicating CN and CrzA-independent growth inhibition by CN585.

### 3.5. Molecular Docking of CN585 Reveals Differential Potential Binding Sites between A. fumigatus and Human CN

**Figure 5:**
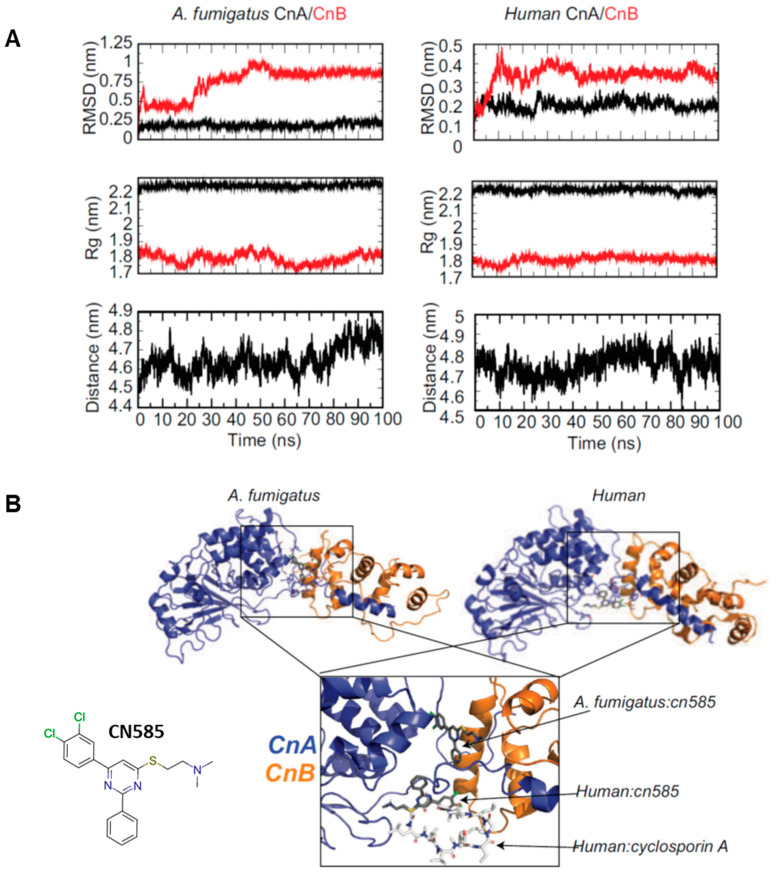
Molecular dynamics simulation and docking of *A. fumigatus* and human CnA/CnB to CN585. (**A**) MD simulation analysis showing the RMSD (top), Rg (middle), and COM of CnA to CnB (bottom) of CnA/CnB during the simulation. (**B**) Docking model of *A. fumigatus* CnA/CnB:CN585 (left—CnA (blue) and CnB (orange) compared to human CnA/CnB:CN585 (right—CnA (blue) and CnB (orange). Overlay of the docking poses of CN585 and CsA is shown at the bottom. Labels provided for clarity.

Next, to structurally characterize a possible molecular interaction of CN585 with *A. fumigatus* and *human* CN we utilized a complex usage of MD simulations and molecular docking protocols. This method ensured a more accurate description of the molecular dynamics a protein and small molecule undergo prior to and in complex when in solution compared to the more traditional static docking models. The 100 ns MD simulation showed equilibration after 10 ns of the 100 ns simulation indicative of a well performed MD simulation (Figure 5A). For the most part, the Cα-RMSD, radius of gyration (Rg), and center of mass between (COM) CnA and CnB stabilized after ~10 ns. Interestingly the results show that HADDOCK blind molecular docking predicted two different binding sites for CN585 when docked against *A. fumigatus* and human CN (Figure 5B). When docked to human CN, CN585 overlays with the binding site of CsA and solely interacts with CnA (Figure 5B right), whereas when docked to *A. fumigatus* CN585 bridges an interface between CnA and CnB at a site allosteric to the known CsA binding site (Figure 5B left).

To supplement the docking analysis, target prediction for CN585 was performed using chemoinformatics resources including the Similarity Ensemble Approach (SEA) [41] and Swiss Target Prediction (STP) [42,43]. These methods yielded predictions based on Tanimoto coefficient similarity to known ligands of a biological target. The results of FK506 and CN585 were analyzed for prediction of interactions with CN or FKBPs. As expected, FK506 did show predicted interaction with FKBPs (Figures S3 and S4), whereas no molecules with high Tanimoto coefficient similarity to CN585 were found to interact with CN or FKBPs (Figure S5). The STP of CN585 also does not suggest any interactions with any phosphatase or any FKBP (Figure S6). Interestingly CN585 showed molecular similarity to ligands of a eukaryotic translation initiation factor 4E-binding protein (eIF4E) and other proteins.

### 3.6. Molecular Modeling and Docking of CN585 Reveals Similar Potential Binding Sites between A. fumigatus and Human eIF4E:m^7^GTP

**Figure 6:**
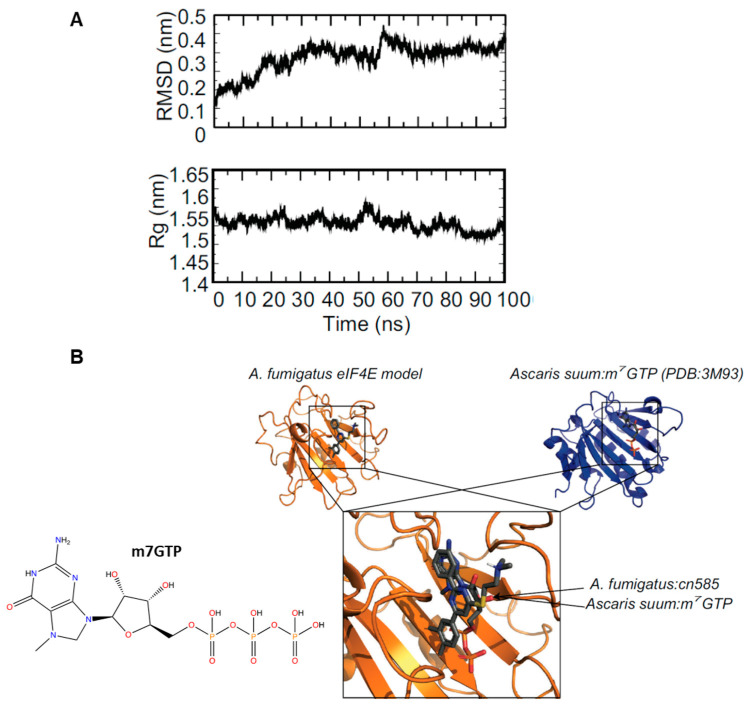
Molecular dynamics simulation and docking of *A. fumigatus* eIF4E to CN585. (**A**) MD simulation analysis showing the RMSD (top) and Rg (middle) of *A. fumigatus* eIF4E during the simulation. (**B**) Docking model of *A. fumigatus* eIF4E: CN585 (orange—left) compared to *Ascaris suum* eIF4E:m^7^GTP (blue—right). Overlay of the docking poses of CN585 and m^7^GTP is shown at the bottom. Labels provided for clarity.

Next to explore a possible interaction of CN585 with eIF4E we first performed the molecular modeling of *A. fumigatus* eIF4E which resulted in a model that overlaid with *Ascaris suum* eIF4E:m^7^GTP (3M93) with a Cα-RMSD of 1.7 Å. To structurally characterize the molecular interaction of CN585 with *A. fumigatus* eIF4E we utilized the same MD simulation and molecular docking protocol as above. The 100 ns MD simulation showed equilibration after 10 ns of the 100 ns simulation indicative of a well performed MD simulation (Figure 6A)—the Cα-RMSD and Rg stabilized after 10 ns. The HADDOCK blind docking results predicted an identical binding site as m^7^GTP with *A. suum* eIF4E (Figure 6B). When docked to *A. fumigatus* eIF4E, CN585 overlays with the binding site of m^7^GTP; however, while in slightly different orientation the aromatic moieties of both CN585 and m^7^GTP overlay in nearly identical orientations.

## 4. Discussion

Due to high structural and conformational similarity between the active site of CnA and other serine/threonine protein phosphatases, other low-molecular weight inhibitors of these phosphatases are also known to inhibit CN [44]. To better understand fungal CN inhibitory mechanisms, here we analyzed the activity of a novel mammalian CN inhibitor, CN585, on *A. fumigatus*. CN585 belongs to a new class of CN inhibitors and characterized as an immunosuppressive and non-competitive reversible inhibitor of CN in mammalian cells [22].

FK506 and CsA are known to inhibit CN by binding to their respective immunophilins FKBP12 and CypA [14,15]. Unexpectedly, we found that in contrast to FK506 the fungal growth inhibition pattern by CN585 was completely different from the usual stunted and hyperbranching phenotype observed upon CN inhibition by FK506 or CsA (Figure 1). For generalizability, we found that the *A. fumigatus* wild-type, azole- and echinocandin-resistant strains, and the various CN mutants showing resistance to FK506/CsA, were equally sensitive to CN585 (Figure 2). As in vitro susceptibility assays with the various CN pathway mutants indicated an unexpected pattern of growth inhibition by CN585, we speculated if CN585 was indeed specifically inhibiting CN function in *A. fumigatus*. These initial findings indicated that CN585 may probably inhibit CN and also other targets in the cell to bring about complete growth inhibition not normally observed with FK506/CsA treatment. While the CN catalytic subunit deletion strain (Δ*cnaA*) exhibited hypersensitivity to CN585 in comparison to FK506 (Figure 3), the nuclear localization of the CN-dependent transcription factor CrzA was also not blocked by CN585 (Figure 4), indicating the possibility of it being non-specific and having off-target effects in *A. fumigatus* leading to antifungal activity. Furthermore, as we have established the requirement of the calcineurin regulatory subunit, CnB, for the function of CnA and shown the phenotypic similarity between the CnA deletion and the CnB deletion strains [38], we expect that the CnB deletion strain strain would also exhibit similar susceptibility to CN585. Taken together, these findings revealed that CN585 also does not bind to the immunophilins to inhibit CN, but may inhibit CN through another alternative mechanism, interaction with other yet unknown residues in CnA, or interaction with other yet unknown target(s).

Based on the molecular docking assessments of *A. fumigatus* and human CN, CN585 binds to different sites when comparing docking to *A. fumigatus* and human; however, the docking scores have similar values to one another and compared to binding of FK506 or CsA. Structurally, when CN585 is docked to human CN it binds in a manner almost identical to that of CsA and has many of the same interactions with CnA residues as CsA binding (Figure 5B). Both CN585 and CsA interact with residues L312, Y341, W342, L343, P344 and W352 on human CnA. While the docked CN585 did not interact with CnB, CsA is known to interact with CnB in a limited manner. The list of residues that interact with CN585 in the docked model are remarkably similar to residues shown as essential to confer resistance to CsA (but not FK506) [45]. Specifically, V314R and Y341F mutations have shown to confer resistance to CsA, while modification of CsA has been proposed to take advantage to include a hydrogen bond donor for the carbonyl of W342 and/or L312 [46]. Interestingly though, the molecular docking of CN585 suggested a completely different site of binding for CN585 when docked to CN from *A. fumigatus* (Figure 5A-left). This docking suggested the *A. fumigatus* binding site is in the interface of CnA and CnB allosteric to the known FK506 or CsA binding site, approximately 16 Å away. There is only one conserved contact with CN585 docking to *A. fumigatus* CN (W364) observed in the docking with human CN, W342. However, we did not observe any CN585 resistance in our W364A mutant (Figure 2). The rest of the contacts in human CN were with residues A21-R53 and D346 of CnA and residues M118, E138 and N158 of CnB. As the docked CN585 structure does not have many similar CsA contacts, it is possible the binding site observed in the *A. fumigatus:*CN585 model would not sterically inhibit the binding of FK506 and/or CsA, therefore in this conformation cannot be directly attributed to inhibitory phenotypes and/or the allosteric binding site. Based on this we hypothesize that CN585 does not in actuality interact with *A. fumigatus* CN.

Interestingly, we also did not find FKBPs as potential interactors of CN585 using the chemoinformatics resources SEA or STP. While FK506 was predicted to interact with FKBPs, no molecules with high Tanimoto coefficient similarity to CN585 were found to interact with CN or FKBPs. Ultimately, neither the structure-based nor ligand-base techniques support a hypothesis of CN585 directly interacting with *A. fumigatus* CN. The computational method utilized here still leaves the possibility that CN585’s antifungal properties may be due to its potential interaction with other proteins that are important for fungal growth.

As such, CN585 was molecularly docked to a model of the eIF4E sequence from *A. fumigatus* (the top hit from STP) to compare its predicted binding site in comparison with known X-ray characterized structure of eIF4E in complex with m^7^GTP in an identical manner to CN585 docking to *A. fumigatus* and *Human* CN. The docking showed a near identical binding site sharing many of the similar/identical residues between the *A. fumigatus* and *Ascaris suum* sequences (Figure 6B). Despite the differences in the chemical composition between m^7^GTP and CN585, these two structures share similar contacts (Figure 6B). *A. fumigatus* eIF4E:CN585 had contacts with Y157, P201, V202, W203 and R254 which are similar to contacts between *Ascaris suum*:m^7^GTP W69, P113, M114, W115 and R170 when the sequences are aligned (Figure S7).

While CN585 has been shown to inhibit mammalian CN [22], our results described here suggested that CN585 does not appear to specifically inhibit *A. fumigatus* CN and caution should be exercised in utilizing CN585 as a CN-specific inhibitor in fungi. Further studies are required to understand the exact mode of inhibition of fungal growth by CN585.

## Supplementary Materials

The following supporting information can be downloaded at: https://www.mdpi.com/article/10.3390/jof8121281/s1. Figure S1: CnaA mutants showing CsA resistance. Figure S2: Generation of *cypA* deletion verified by Southern analysis and susceptibility to CsA. Figure S3: Swiss Target Prediction for FK506. Figure S4: Similarity Ensemble Approach prediction of FK506. Figure S5: Swiss Target Prediction for CN585. Figure S6: Similarity Ensemble Approach prediction of CN585. Figure S7: Alignment of *A. fumigatus* and *Ascaris suum* eIF4.
