# Supplementary material for: Calcineurin Inhibitor CN585 Exhibits Off-Target Effects in the Human Fungal Pathogen Aspergillus fumigatus
Source: J Fungi (Basel). 2022 Dec 7;8(12):1281. doi: 10.3390/jof8121281 (PMC9788591; doi:10.3390/jof8121281)
Supplement: Supplementary file 1 [file jof-08-01281-s001.zip › jof-2076690-supplementary.pdf]

Figure S1

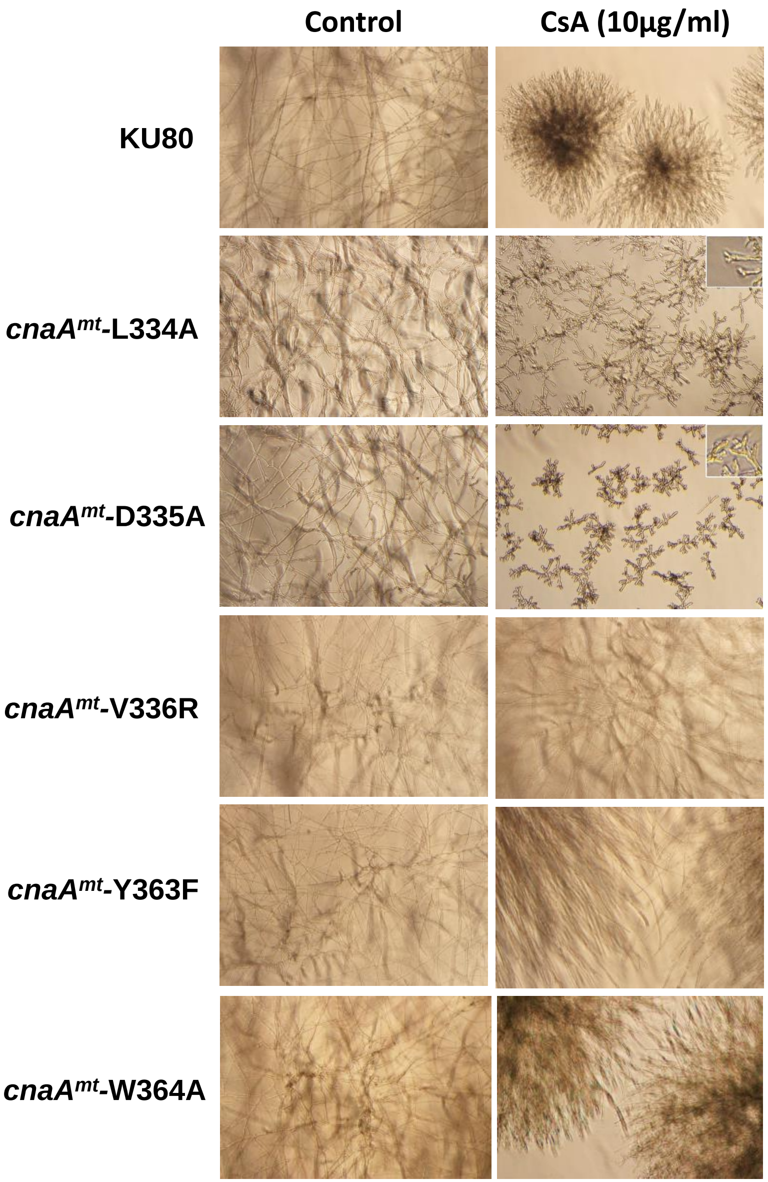

Figure S1. Growth of the wild-type *A. fumigatus* (KU80) and the *cnaA* mutant strains in the presence of CsA. DMSO control growth is shown. Growth was monitored for 2 days in RPMI at 37˚C. Note the resistance at 10 μg/ml CsA in the mutants. Growth susceptibility assays were performed three times in triplicate.

Figure S2

Generation of *cypA* deletion verified by Southern analysis and susceptibility to CsA

A

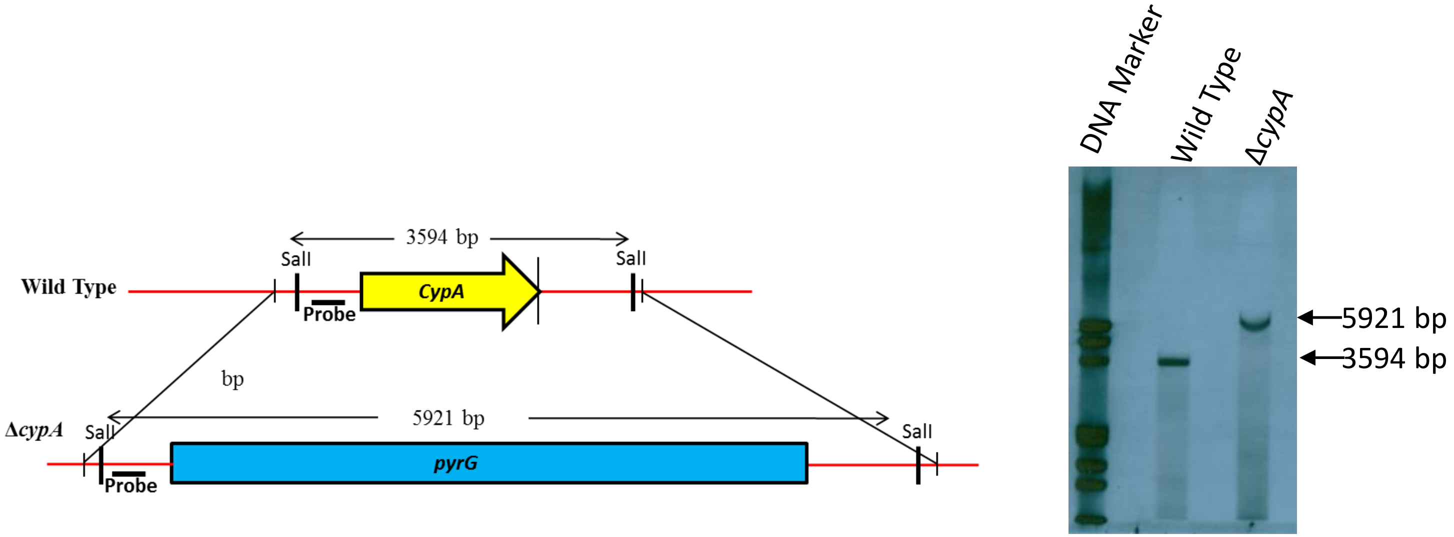

B

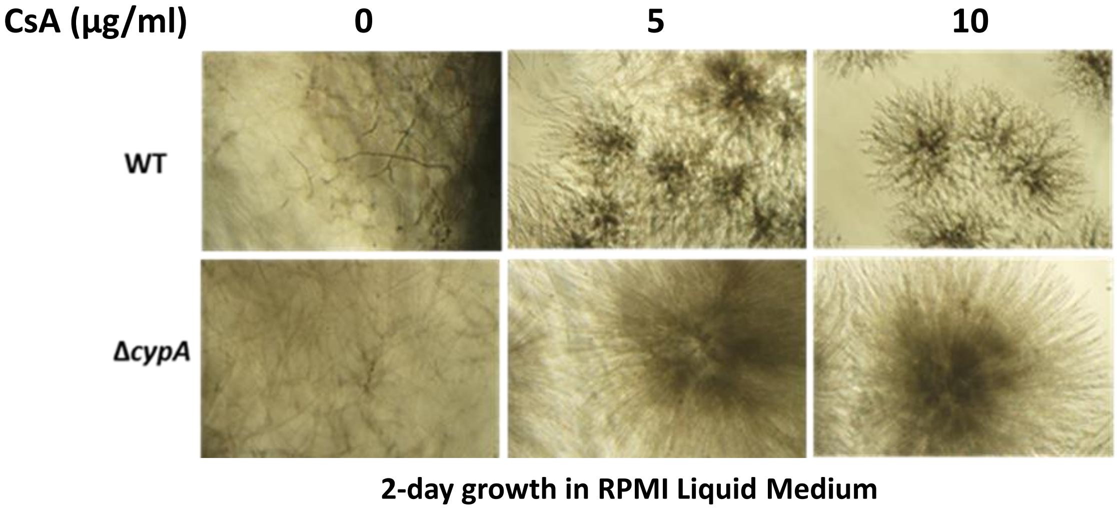

Figure S2. A. Southern analysis for the verification of the deletion of the cyclophilin A gene *cypA* in *A. fumigatus* (KU80). **B.** Growth of the *cypA* mutant strain in the presence of CsA. Growth was monitored for 2 days in RPMI at 37˚C. Note the resistance at 5 and 10 µg/ml CsA in the *cypA* mutant in comparison to the wild-type strain. Growth susceptibility assays were performed three times in triplicate.

Figure S3

Swiss Target Prediction for FK506

Target	Common name	Uniprot ID	ChEMBL ID	Target Class	Probability*	Known actives (3D/2D)
Splicing factor 3B subunit 3	SF3B3	Q15393	CHEMBL1250378	Unclassified protein	1.0	8 / 2
FK506-binding protein 1A	FKBP1A	P62942	CHEMBL1902	Isomerase	1.0	79 / 129
Peptidyl-prolyl cis-trans isomerase FKBP5	FKBP5	Q13451	CHEMBL2052031	Enzyme	1.0	19 / 4
FK506-binding protein 1B	FKBP1B	P68106	CHEMBL2430	Enzyme	1.0	2 / 2
Serine/threonine-protein kinase mTOR	MTOR	P42345	CHEMBL2842	Kinase	1.0	182 / 5
Cytochrome P450 3A4	CYP3A4	P08684	CHEMBL340	Cytochrome P450	1.0	20 / 3
Serine/threonine protein phosphatase 2B catalytic subunit, alpha isoform	PPP3CA	Q08209	CHEMBL4445	Phosphatase	1.0	11 / 15
FK506 binding protein 4	FKBP4	Q02790	CHEMBL4050	Enzyme	0.581005955019	13 / 2

Swiss Target Prediction program was used to analyze the targets for FK506

Figure S4

Similarity Ensemble Approach prediction of FK506

Query	Target Key	Target Name	Description	P-Value	MaxTC
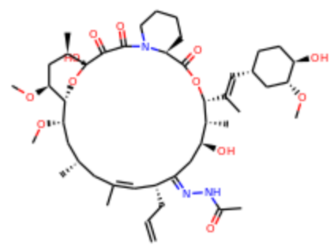 compound_1	PP2BA_HUMAN	PPP3CA	Serine/threonine-protein phosphatase 2B catalytic subunit alpha isoform	1.214e-192	0.83
	FKB1A_HUMAN	FKBP1A	Peptidyl-prolyl cis-trans isomerase FKBP1A	4.884e-143	0.83
	FKB1A_MOUSE	Fkbp1a	Peptidyl-prolyl cis-trans isomerase FKBP1A	1.543e-105	0.38
	FKB1B_HUMAN	FKBP1B	Peptidyl-prolyl cis-trans isomerase FKBP1B	1.013e-59	0.83
	SO1B1_HUMAN	SLCO1B1	Solute carrier organic anion transporter family member 1B1	1.304e-23	0.83
	SF3B3_HUMAN	SF3B3	Splicing factor 3B subunit 3	4.796e-22	0.83
	FKBP4_HUMAN	FKBP4	Peptidyl-prolyl cis-trans isomerase FKBP4	3.026e-13	0.74
	PDCD4_HUMAN	PDCD4	Programmed cell death protein 4	1.713e-10	0.35
	FKBP5_HUMAN	FKBP5	Peptidyl-prolyl cis-trans isomerase FKBP5	1.73e-09	0.83

Similarity Ensemble Approach prediction program was used to analyze the targets for FK506

Figure S5

Swiss Target Prediction for CN585

Target	Common name	Uniprot ID	ChEMBL ID	Target Class	Probability*	Known actives (3D/2D)
Norepinephrine transporter	SLC6A2	P23975	CHEMBL222	Electrochemical transporter	0.109339753231	724 / 0
Serine/threonine-protein kinase PIM1	PIM1	P11309	CHEMBL2147	Kinase	0.109339753231	373 / 0
Histamine H4 receptor	HRH4	Q9H3N8	CHEMBL3759	Family A G protein-coupled receptor	0.109339753231	409 / 0
Urotensin II receptor	UTS2R	Q9UKP6	CHEMBL3764	Family A G protein-coupled receptor	0.109339753231	127 / 0
Dopamine D4 receptor	DRD4	P21917	CHEMBL219	Family A G protein-coupled receptor	0.109339753231	666 / 0
Neuronal acetylcholine receptor protein alpha-4 subunit	CHRNA4	P43681	CHEMBL1882	Ligand-gated ion channel	0.109339753231	16 / 0
Histamine H3 receptor	HRH3	Q9Y5N1	CHEMBL264	Family A G	0.109339753231	1125 / 0

Swiss Target Prediction program was used to analyze the targets for CN585

Figure S6

Similarity Ensemble Approach prediction of CN585

Query	Target Key	Target Name	Description	P-Value	MaxTC
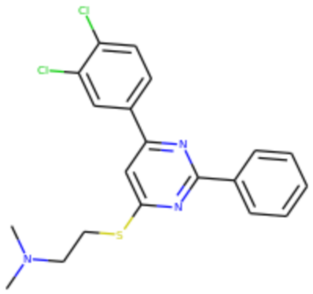 compound_1	IF4E_HUMAN	EIF4E	Eukaryotic translation initiation factor 4E	6.785e-43	0.33
	NK2R_BOVIN	TACR2	Substance-K receptor	1.328e-30	0.32
	UR2R_HUMAN	UTS2R	Urotensin-2 receptor	4.831e-22	0.34
	LIPS_RAT	Lipe	Hormone-sensitive lipase	2.809e-14	0.32
	KMO_HUMAN	KMO	Kynurenine 3-monooxygenase	3.586e-14	0.35
	AHR_MOUSE	Ahr	Aryl hydrocarbon receptor	6.449e-13	0.28
	TKNK_HUMAN	TAC3	Tachykinin-3	1.532e-12	0.31
	NK2R_RAT	Tacr2	Substance-K receptor	5.158e-12	0.32
	KMO_MOUSE	Kmo	Kynurenine 3-monooxygenase	6.139e-12	0.29
	NK2R_HUMAN	TACR2	Substance-K receptor	4.694e-11	0.33
	LIPS_HUMAN	LIPE	Hormone-sensitive lipase	4.201e-09	0.32
	DXR_MYCTU	dxr	1-deoxy-D-xylulose 5-phosphate reductoisomerase	7.045e-07	0.31
	NORA_STAAU	norA	Quinolone resistance protein NorA	1.614e-06	0.31
	5HT7R_HUMAN	HTR7	5-hydroxytryptamine receptor 7	2.502e-06	0.52
	RAD52_HUMAN	RAD52	DNA repair protein RAD52 homolog	2.937e-06	0.34

hologs/AHR_MOUSE

Similarity Ensemble Approach prediction program was used to analyze the targets for CN585

Figure S7

Alignment of *A. fumigatus* and *Ascaris suum* eIF4

```
                       10        20        30        40        50        60
                        |         |         |         |         |         |
A. suum      MRHPLQCHWALWYLKAD-RSKDWEDCLKQVAVFDTVEDFWSLYNHIQAASGLTWGSDYYL
A. fum       KEHALKSTWVIWYRPPTPKYSDYEKSTIPLASISSVESFWSIYTHLKRPSLLPTVSDYHI
              .*.*:. *.:**  .  : .*:*..   :* :.:**.***:*.*:: .* *.  ***::

Prim.cons.   22H2L222W22WY2222P222D2E222222A2222VE2FWS2Y2H2222S2L222SDY22

                       70        80        90       100       110       120
                        |         |         |         |         |         |
A. suum      FKEGIKPMWEDENNVKGGRWLVVVDKQKRAQLLDHYWLELLMAIIGEQFEDNGEYICGAV
A. fum       FKKGIRPVWEDDANKKGGKWIVRLKKG----VADRYWEDLLLAMIGDQFAEASDEVCGAV
             **:**:*:***: * ***:*:* :.*     : *:** :**:*:**:** : .: :****

Prim.cons.   FK2GI2P2WED22N2KGG2W2V222K2KRAQ22D2YW22LL2A2IG2QF2222222CGAV

                      130       140       150       160       170       180
                        |         |         |         |         |         |
A. suum      VNVRQKGDKVSLWTRDSLKDDVNLRIGQILKAKLEIPDTEPIRYEVHKDSSVRTGSMVKP
A. fum       LSVRSGEDVLSVWTR--IDGGRNIKIRETIKRLLAFPADTNIVWKSHDDSIAQRSAIDQA
             :.**.  * :*:***  :... *::* : :*  * :*    * :: *.** .: .:: :.

Prim.cons.   22VR222D22S2WTRDS22222N22I2222K22L22P2222I2222H2DS2222222222

                      190
                        |
A. suum      RIVIPSKDNR
A. fum       RQ--------
             *

Prim.cons.   R2VIPSKDNR
```
